# Supplementary material for: A Critical View of the Application of the APEX Software (Aqueous Photochemistry of Environmentally-Occurring Xenobiotics) to Predict Photoreaction Kinetics in Surface Freshwaters
Source: Molecules. 2019 Dec 18;25(1):9. doi: 10.3390/molecules25010009 (PMC7017383; doi:10.3390/molecules25010009)
Supplement: Supplementary file 1 [file molecules-25-00009-s001.zip › 3.2.4_gcc-4.4.0.zip!/3.2.4_gcc-4.4.0/3.2.4_gcc-4.4.0/doc/octave/html/interpreter/Creating-Packages.html]

Creating Packages - Untitled


Previous: Administrating Packages,
Up: Packages


---

### 35.4 Creating Packages

Internally a package is simply a gzipped tar file that contains a
top level directory of any given name. This directory will in the
following be referred to as `package` and may contain the
following files

`package/DESCRIPTION`: This is a required file containing information about the package. See The DESCRIPTION File, for details on this file. `package/COPYING`: This is a required file containing the license of the package. No restrictions is made on the license in general. If however the package contains dynamically linked functions the license must be compatible with the GNU General Public License. `package/INDEX`: This is an optional file describing the functions provided by the package. If this file is not given then one with be created automatically from the functions in the package and the `Categories` keyword in the `DESCRIPTION` file. See The INDEX file, for details on this file. `package/PKG_ADD`: An optional file that includes commands that are run when the package is added to the users path. Note that `PKG_ADD` directives in the source code of the package will also be added to this file by the Octave package manager. Note that symbolic links are to be avoided in packages, as symbolic links do not exist on some file systems, and so a typical use for this file is the replacement of the symbolic link ``` ln -s foo.oct bar.oct ``` with an autoload directive like ``` autoload ('bar', which ('foo')); ``` See PKG\_ADD and PKG\_DEL directives, for details on `PKG_ADD` directives. `package/PKG_DEL`: An optional file that includes commands that are run when the package is removed from the users path. Note that `PKG_DEL` directives in the source code of the package will also be added to this file by the Octave package manager. See PKG\_ADD and PKG\_DEL directives, for details on `PKG_DEL` directives. `package/pre_install.m`: This is an optional script that is run prior to the installation of a package. `package/post_install.m`: This is an optional script that is run after the installation of a package. `package/on_uninstall.m`: This is an optional script that is run prior to the removal of a package.

Besides the above mentioned files, a package can also contain on or
more of the following directories

`package/inst`: An optional directory containing any files that are directly installed by the package. Typically this will include any `m`-files. `package/src`: An optional directory containing code that must be built prior to the packages installation. The Octave package manager will execute ./configure in this directory if this script exists, and will then call `make` if a file Makefile exists in this directory. `make install` will however not be called. If a file called `FILES` exists all files listed there will be copied to the `inst` directory, so they also will be installed. If the `FILES` file doesn't exist, src/\*.m and src/\*.oct will be copied to the `inst` directory. `package/doc`: An optional directory containing documentation for the package. The files in this directory will be directly installed in a sub-directory of the installed package for future reference. `package/bin`: An optional directory containing files that will be added to the Octave `EXEC_PATH` when the package is loaded. This might contain external scripts, etc., called by functions within the package.

- The DESCRIPTION File- The INDEX file- PKG\_ADD and PKG\_DEL directives
